# Significance and implications of *FHIT* gene expression and promoter hypermethylation in acute lymphoblastic leukemia (ALL)

**DOI:** 10.1007/s12672-024-00971-9

**Published:** 2024-04-08

**Authors:** Fozia Mohammad, Arshad A. Pandith, Shayaq Ul Abeer Rasool, Faisal R. Guru, Iqbal Qasim, Sajad Geelani, Syed Nisar, Shahid M. Baba, Farooq A. Ganie, Safiya Kouser, Javid Rasool

**Affiliations:** 1 Advanced Centre for Human Genetics, Sher-I-Kashmir Institute of Medical Sciences (SKIMS), Srinagar, J&K 190011 India; 2 Department of Medical Oncology, SKIMS, Srinagar, J&K, 190011 India; 3 School of Life Sciences, Jaipur National University, Jaipur, Rajasthan 302017 India; 4 Department of Hematology, SKIMS, Srinagar, 190011 J&K India; 5 Department of Urology, SKIMS, Srinagar, 190011 J&K India; 6 Department of CVTS, SKIMS, Srinagar, 190011 J&K India

**Keywords:** Acute lymphoblastic leukemia, *FHIT* expression, *FHIT* gene hypermethylation, B-cell ALL, mRNA expression

## Abstract

**Background:**

Fragile histidine triad (*FHIT*) has been documented to play a vital role in various cancers including acute lymphoblastic leukemia (ALL). Keeping in view the plausible role of *FHIT* gene, we aimed to examine DNA promoter hypermethylation and mRNA expression in ALL cases in Kashmir (North India).

**Methods:**

A total of 66 cases of ALL were analyzed for *FHIT* mRNA expression and promoter methylation by qRT-PCR and Methylation Specific-PCR (MS-PCR) respectively.

**Results:**

*FHIT* mRNA expression showed significantly decreased expression in ALL cases with mean fold change of 9.24 ± 5.44 as compared to healthy controls (p = 0.01). The pattern of *FHIT* deregulation in ALL cases differed significantly between decreased and increased expression (p < 0.0001). A threefold decreased expression was observed in 75% of ALL cases than healthy controls (− 3.58 ± 2.32). ALL patients with *FHIT* gene promoter hypermethylation presented significantly higher in 80% (53/66) of cases (p = 0.0005). The association of *FHIT* gene hypermethylation and its subsequent expression showed *FHIT* mRNA expression as significantly lower in ALL cases with hypermethylation (*p* = 0.0008). B-ALL cases exhibited a highly significant association between the methylation pattern and its mRNA expression (p = 0.000). In low range WBC group, a significant association was found between increased expression (26%) of the cases and methylated (4%)/unmethylated group 86% (p = 0.0006).

**Conclusion:**

The present study conclude that *FHIT* gene hypermethylation and its altered expression may be linked in the pathogenesis of ALL and provide an evidence for the role of *FHIT* in the development of ALL.

**Supplementary Information:**

The online version contains supplementary material available at 10.1007/s12672-024-00971-9.

## Introduction

Among different types of leukemia, acute lymphoblastic leukemia (ALL) is the most common form of childhood cancer. As per Surveillance, Epidemiology, and End Results (SEER) program, ALL accounts for ~ 25% of all diagnosed cancers in children up to the age group of 14 years [1]. Here in this region (J&K, India), ALL ranks 5th among most common cancers and occurs at a frequency of 9.9% and the average incidence of leukemia is 5.8/10^5^/year with highest incidence in ALL cases [2]. As per the latest report, Leukemia is the most prevalent childhood cancer that accounts for 36.6% of cases and ALL was the most common type found than any other subtypes [3]. While 80% of ALL occurs in children, it is a more devastating disease in adults. Among ALL, Precursor B-cell ALL (BCP-ALL) represents 73–85% of total cases. B-ALL is generally related with a better outcome in children where the rate of cure touches ~ 90% [4]. Despite refinement for improved treatment response, ALL still poses a challenge as a leading cause of cancer-related mortality due to relapse [5]. Several studies have investigated the progression of genetic alterations from diagnosis to relapse that contribute to drug resistance and the assumption is that identifying the basic biological mechanisms that are accountable for drug resistance/relapse would offer rational resources for both inhibition of relapse and successful salvage therapy [6, 7]. Analyses of DNA copy number mutations, gene expression, DNA methylation, and next-generation sequencing of ALL samples in addition to chromosomal aberrations have been used to classify global genetic and epigenetic changes that characterize disease progression [8–11]. There are specific established risk factors in ALL that primarily include gender, age, performance score, leukocyte count, cytogenetics and molecular alterations and the immunophenotyping etc. ([12]). Chromosomal aberrations are common characteristics in ALL and the fragile histidine triad (*FHIT*) gene is a main spot of chromosomal rearrangement [13]. The *FHIT* gene acts as tumor suppressive gene and its tumor suppressive properties have been shown to be restored by transfection of *FHIT* in *FHIT*‑deficient human cancer cells that emerge to stimulate apoptosis and hamper cellular growth [14, 15]. *FHIT* promoter hypermethylation and its expression has been observed as one of the key events in the pathogenesis of ALL. Promoter DNA methylation, an epigenetic modification is notably seen to contribute to the development of leukemia as confirmed in case of *FHIT* gene in various forms of cancers [16–18] and in particular ALL [19, 20]. Pathogenesis of many leukemic disorders has been associated with *FHIT* loss of function as a consequence of its down regulation or aberrant expression [21]. In ALL, loss of *FHIT* expression causes inactivation of *FHIT* protein that may result in the development of leukemias [22]. Keeping in view the plausible role of *FHIT* gene, we conducted this study to observe the pattern and significance of *FHIT* gene promoter methylation and its expression in the pathogenesis of ALL with respect to clinic-pathological characteristics.

## Methods

The present study was conducted in Advanced Center for Human Genetics, Sher-i-Kashmir institute of Medical Sciences (SKIMS), Srinagar (J&K, India). A total of 66 ALL patients were recruited between 2018 and 2021 who attended the Department of Clinical Hematology and Medical Oncology (SKIMS).. The diagnosis of ALL was confirmed by clinical assessment along with bone marrow and cytochemical cytopathology. ALL patients were categorized according to the French & British American classification (FAB) and WHO classification of acute leukemia. ALL patients were classified into different set of risk groups and treatment was given as per the modified BFM-95 protocol [23]. Peripheral blood samples (2–3 ml) were taken from all the patients and from age and sex matched healthy controls with no signs of any hematological/and or any other malignancy from the Out Patients Department of SKIMS. The samples were immediately extracted for DNA and RNA analysis and stored at – 80 ℃ for further processing. The local ethics committee of SKIMS approved the study and written informed consent was obtained from cases (parent consent in case of minor) as well as controls. All patients and guardians were told about the research protocol and blood samples were taken only after signing a previous approval by their patients or guardians. Data of ALL patients (medical records and hematology/bone marrow reports) was obtained from personal interviews with patients and or guardians.

### DNA extraction 

Blood samples from ALL patients were used for DNA extraction through phenol/chloroform method and/or DNA extraction kit (Qaigen, Germany). DNA concentration was estimated for purity at the absorbance of 260/280 nm and verified on 0.8–1% agarose gel.

### Analysis of FHIT gene hypermethylation by MS-PCR

Around 1.5–2 µg of DNA extracted was modified with bisulfite modification treatment (EZ-DNA Methylation Kit, Zymo Research Corporation, USA). A methylation specific-PCR (MSP-PCR) was done as per the protocol published by Golam Reza et al. [24]. The gel pictures were analyzed for bands by gel documentation system Flourchem HD2 (Cell Bioscience, USA).

### Quantitative real-time reverse transcription polymerase chain reaction (qRT-PCR) for *FHIT m*RNA analysis

From fresh blood samples using TRIZOL reagent (Ambion, Thermo Fisher, USA), RNA was extracted as per the manufacturer’s protocol. The concentration of RNA was examined using a UV–Vis Spectrophotometer (Thermo Scientific,Nano Drop 2000) with a wavelength of 260 nm. Integrity of RNA was checked using 1% Agarose gel (Additional file 1: Figure S1). The DNase, RNase- free (Thermo Scientific, USA) reagent kit was used to remove the traces of genomic DNA from RNA preparation and the Thermo Scientific^™^ RevertAid^™^ First Strand cDNA Synthesis Kit (Thermo Scientific, USA) was used for cDNA synthesis as per the protocol using primers as shown in Additional file Table S1. For qRT-PCR of *FHIT m*RNA expression, the PCR amplification was performed on Rotor-Gene Q accessories (Qiagen, Germany) using Maxima SyBR Green /ROX qPCR Master Mix (2x) with an internal control gene GAPDH as per the protocol (primers: Additional file Table S1). The cycle threshold (Ct) was analyzed and fold changes of gene of interest relative to GAPDH were done by the formula of 2^−ΔΔCt^. The results were finally presented as an average log2 fold change which represented a decrease or increase in the mRNA expression of the respective gene.

**Table 1:** Expression of FHIT mRNA with respect to different clinico-pathological parameters

Parameters	ALL cases	*FHIT* mRNA expression (mean ± SD)	95% CI	p value
Gender				
Male	18 (67.5%)	− 2.64 ± 2.32		0.013
Female	12 (32.5%)	− 4.99 ± 2.51	− 4.179 to − 0.5206	
Age				
≤ 18 years	21 (62.5%)	− 3.72 ± 2.26		0.61
> 18 years	09 (37.5%)	− 3.25 ± 2.54	− 1.432 to 2.392	
WBC counts (10^9^/L)				
< 10	20 (65%)	− 3.71** ± **2.67		0.68
≥ 10	10 (35%)	− 3.33 ± 1.70	1.525 to 2.285	
Immunophenotype precursor-B				
ALL	26 (97.5%)	− 3.69 ± 2.24		0.5
T-ALL	04 (2.5%)	− 2.90 ± 3.05	− 1.785 to 3.365	
Platelet cells/mm^3^				
≥ 150	09 (22.5%)	− 5.06 ± 2.50		0.05
< 150	21 (77.5%)	− 3.21 ± 2.17	− 0.0018 to 3.702	
Hemoglobin concentration (g/dL)				
< 10	23 (77%)	− 3.3 ± 2.33	− 3.231 to 0.8308	0.23
≥ 10	07 (23%)	− 4.5 ± 2.17	− 3.231 to 0.8308	
Total no. of patients n = 66(%)	FHIT promoter methylation			
	Methylated	Unmethylated		0.0005
	n = 53(80%)	n = 13(20%)		

### Statistical analysis

Statistical analysis for the results of the study was done using IBM Statistics SPSS software, version-23 (IBM Corp, NY, USA). For clinico-pathological characteristics, categorical variables (gender and age) in case and controls were matched using chi-squared test. Expression analysis *FHIT* gene was expressed as cycle threshold values. Comparative analysis was performed Livak Method (^ΔΔ^Ct) where data interpretation was exhibited in terms of relative fold expression.

## Results

*FHIT* gene methylation was conducted by MS-PCR (n = 66) while as analysis of *FHIT* mRNA expression levels were conducted in corresponding 40 cases of ALL (25 males and 15 females; mean age, of 12.2 ± 3.4 years; with an age range, of 1–27 years) and 40 healthy (age and sex matched) children 30 males and 12 females; mean age, of 12.3 ± 4.1 years; with an age range, of 1–30 years). The clinic-pathological characteristics of the studied subjects are given in Table 1.

ALL patient samples analyzed for *FHIT* gene promoter hypermethylation showed significantly higher frequency of hypermethylation in 80% of cases (53/66) (p = 0.0005; Table 1).

**Fig. 1:**
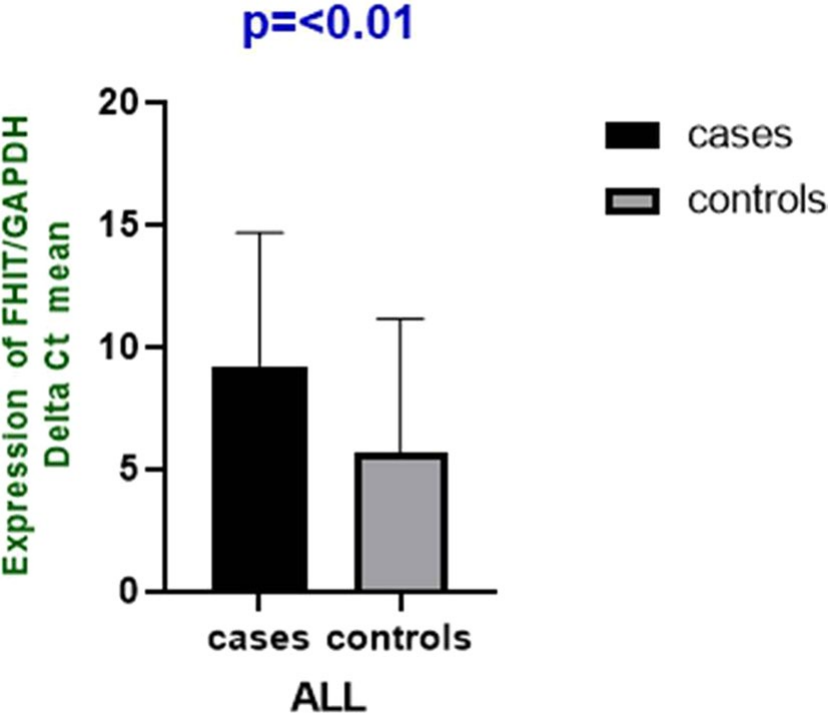
mRNA expression of FHIT gene as determined by qRT-PCR in ALL cases and healthy controls (n = 40)

**Fig. 2:**
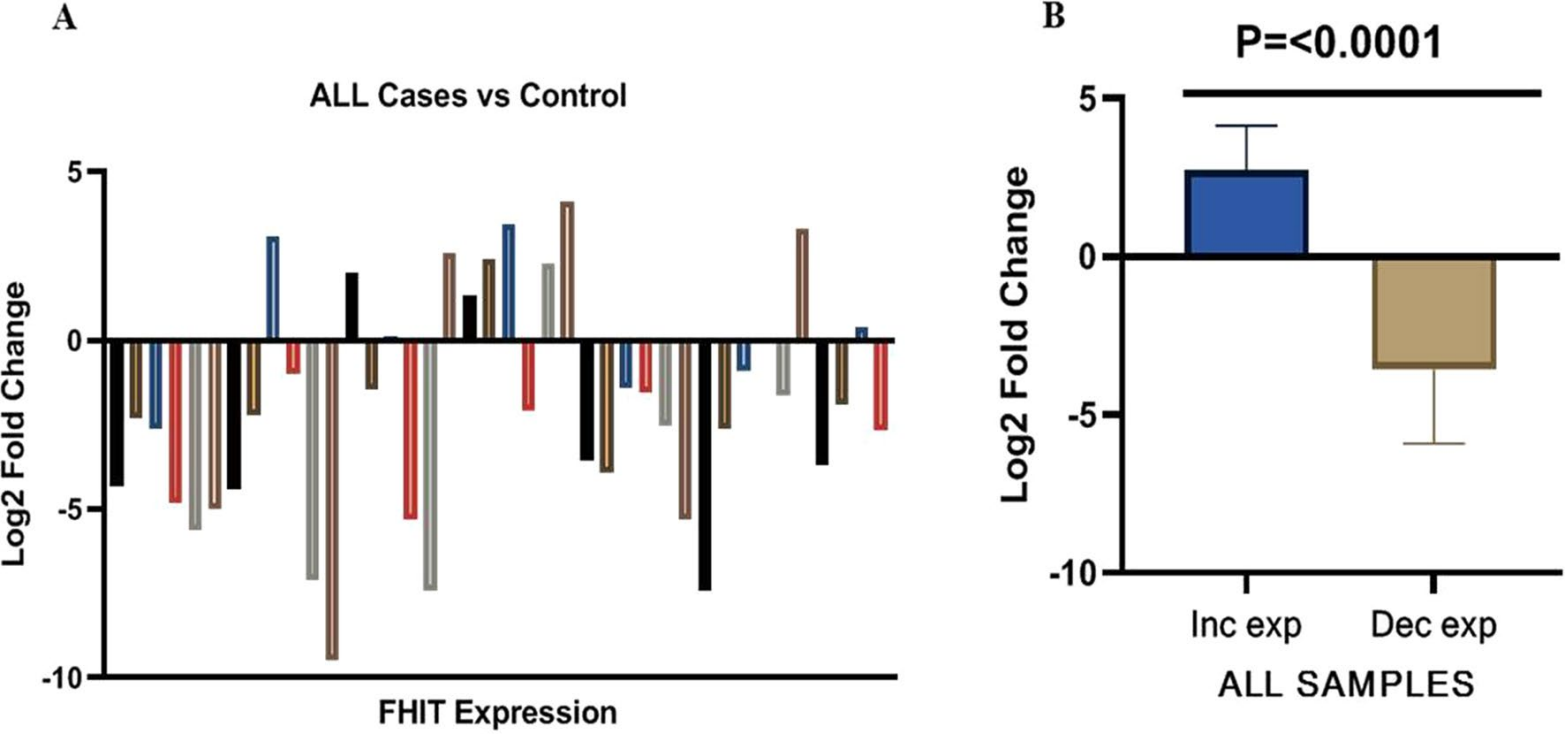
The relative expression analysis of all the cases (**A**) and overall comparison of increased and decreased fold change expression of FHIT gene in ALL cases (**B**)

*FHIT* mRNA expression showed significantly decreased expression in 75% of ALL cases with mean fold change of 9.24 ± 5.44 as compared to healthy controls (p = 0.01: Fig. 1). The pattern of altered lower expression was significantly more in ALL cases than controls (p < 0.0001: Table 2). Relative expression of *FHIT* gene was successfully analyzed in all samples and melt curve analysis was also performed with every experiment which revealed there is stability with no non-specificity within the reaction (Additional file Fig. 2A and B). Lower expression was observed in 75% (30/40) of ALL cases as compared to healthy controls with mean log2 fold change of − 3.58 ± 2.32 that signifies threefold decreased expression (Table 2). Overall 25% (n = 10) cases showed increased expression with an average log2 fold change 2.74 ± 1.40 which signifies 2-fold increased expression of *FHIT* gene in the cases than controls. However, high significance was observed when decreased expression of ALL cases was compared with the cases having increased expression (Fig. 2B). Among the clinico-pathological characteristics of the patients, *FHIT gene* expression depicted significant difference in gender (p < 0.013) and rest of the parameters showed no association (p > 0.05) (Table 1).

**Table 2:** FHIT gene expression expressed as average fold change (Avg. 2^–(ΔΔCt)^) in ALL cases

GENE	Average decreased log2 of fold change in 30 ALL cases	Average increased log2 of fold change in 10 ALL cases	95% CI	P value
	Mean ± SD	Mean ± SD		
FHIT	− 3.58 ± 2.32	2.74 ± 1.40	− 7.901 to − 4.739	< 0.0001

### Correlation of *FHIT* gene promoter hypermethylation with its expression

The association of *FHIT* gene promoter hypermethylation and its subsequent expression were performed on previously obtained samples. The relative expression analysis of all the cases of ALL is shown in Fig. 2A. Of the 30 samples with decreased *FHIT* gene expression, 90% (27/30) were hypermethylated, while increased expression exhibited 20% (02/10) as methylated sequences (Table 3 and Fig. 2B). The results showed that *FHIT* mRNA expression was significantly lower in ALL cases with hypermethylation (*p* = 0.0008). The association of *FHIT* hypermethylation and its expression in different clinical parameters of ALL patients are shown in (Table 3).

**Table 3:** Association of FHITHyper-methylation and its expression in different clinical parameters of ALL patients

Characteristics FHIT expression	No. of samples (n = 40)	Methylated *FHIT*	Unmethylated *FHIT*	p value
Overall cases				
Decreased expression	30 (75%)	27 (90%)	03 (10%)	0.00008
Increased expression	10 (25%)	02 (20%)	08 (80%)	
Gender				
Male				
Decreased expression	18 (45%)	18 (100%)	0 (0)	–
Increased expression	07 (17.5%)	02 (28.5%)	05 (71.5)	
Female				
Decreased expression	12 (30%)	09 (75%)	03 (25%)	–
Increased expression	03 (7.5%)	0 (0)	02 (100%)	
AGE				
≤ 18 years				
Decreased expression	21 (52.5%)	18 (86%)	03 (14%)	0.01
Increased expression	05 (12.5%)	01 (20%)	04 (80%)	
> 18 years				
Decreased expression	09 (22.5%)	08 (89%)	01 (11%)	0.02
Increased expression	05 (12.5%)	01 (20%)	04 (80%)	
WBC counts (10^9^/l) < 10				
Decreased expression	20 (50%)	18 (90%)	02 (10%)	0.0006
Increased expression	07 (17.5%)	01 (14%)	06 (86%)	
WBC counts (10^9^/l) ≥ 10				
Decreased expression	10 (25%)	09 (90%)	01 (10%)	0.10
Increased expression	03 (7.5%)	01 (33%)	02 (67%)	
Immunophenotype				
B ALL				
Decreased expression	26 (65%)	24 (92%)	02 (8%)	0.0000
Increased expression	09 (22.5%)	01 (11%)	08 (89%)	
T-ALL				
Decreased expression	04 (10%)	03 (75%)	01 (25%)	–
Increased expression	01 (2.5%)	01 (100%)	0	
Platelet cells/mm^3^				
≥ 150				
Decreased expression	07 (17.5%)	06 (86%)	01 (14%)	0. 41
Increased expression	02 (5%)	01 (50%)	01 (50/%)	
< 150				
Decreased expression	23 (57.5%)	22 (96%)	01 (4%)	0.0002
Increased expression	08 (20%)	02 (25%)	06 (75%)	
Hb (g/dL) < 10				
Decreased expression	23 (57.5%)	20 (87%)	03 (13%)	0.0004
Increased expression	10 (25%)	2 (20%)	08 (80%)	
Hb (g/dL) ≥ 10				
Decreased expression	07 (17.5%)	07 (100%)	0	–
Increased expression	0	0	0	
Fusion transcript + ve				
Decreased expression	13	11 (85%)	10 (77%)	0.4
Increased expression		2 (15%)	3 (23%)	
Fusion transcript -ve	27			
Decreased expression		19 (70%)	20 (74%)	1.0
Increased expression		8 (30%)	7 (26%)	

**Fig. 3:**
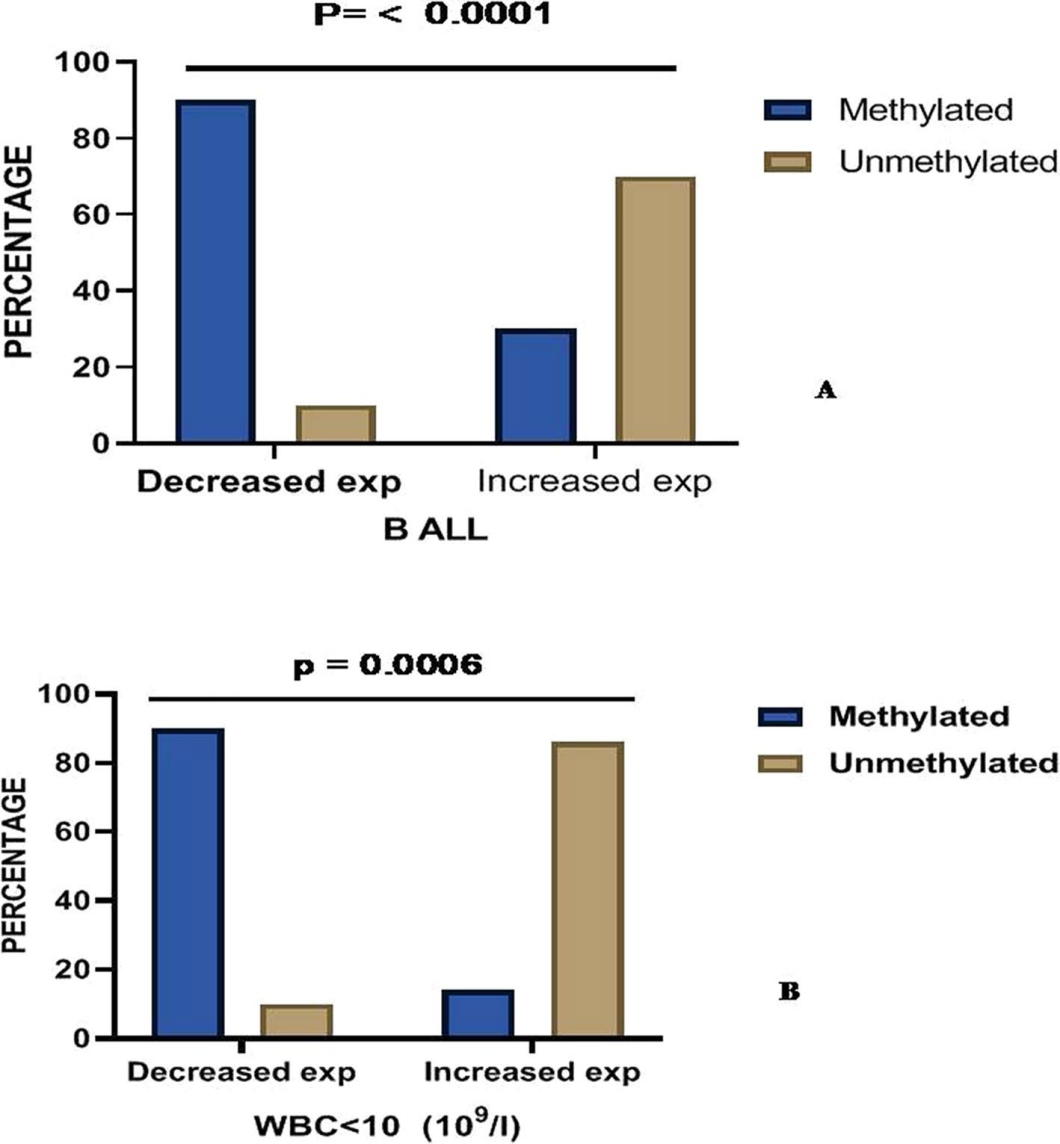
Correlation between FHIT Methylation and its mRNA expression (**A**) and FHIT Methylation and its mRNA Expression with WBC count in ALL patients (**B**)

Among immunophenotypes of ALL, B-ALL showed concordant pattern between hypermethylation and mRNA expression. Decreased *FHIT* expression was observed in 74.2% (26/35) wherein methylated DNA sequences accounted for 68.5% (24/35). Cases with increased mRNA expression of *FHIT* gene was observed in 25.7% (9/35) wherein 2.8% (1/35) showed hypermethylation. In comparison to T-ALL, a highly significant association was observed between the pattern of methylation and its mRNA expression with B-ALL cases (p = 0.000: Fig. 3A).

Among pediatric age group (< 18 years), 86% (18/21) showed decreased *FHIT* mRNA expression and accounted for 81% (21/26) of the methylated cases. Increased *FHIT* expression was detected in 19% (05/26) of the cases with 20% (01/5) methylated cases (Additional file Figure S3).

In low range WBC (< 10 × 109/L) group, *FHIT* expression was decreased in 74% (20/27) of the cases where 90% (18/20) showed hypermethylated sequences. When compared to rest of the cases in low range WBC group, a significant association was observed between increased expression (26%) of the cases and methylated (4%)/unmethylated group (86%) (p = 0.0006: Table 3 and Fig. 3B). *FHIT* mRNA expression level for both lower platelet and higher group of ALL patients were significantly decreased in methylated cases, as compared to increased expression observed in unmethylated cases. In lower platelet groups, highly significant association was observed between the methylation status and its mRNA expression among the ALL cases (p = 0.0002). Fusion transcripts were detected in 32.5% (13/40) of the cases and the frequency of these transcripts were as BCR-ABL (n = 4), ETV-RUNX1 (n = 6), MLL-AF4 (n = 2) and TCF-PBX1 (n = 1). When positive fusion transcripts were correlated with *FHIT* gene hypermethylation and its subsequent expression, 85% (11/13) cases showed decreased expression amongst which 77%(10/13) belong to methylated cases. However, association between the status of fusion transcript cases to expression and methylation did not reveal any significance (Table 3). A Comparison of fold change expression of *FHIT* gene with clinical parameters is depicted in Additional file 1: Figure S3

## Discussion

*FHIT* gene has been associated with a wide range of tumor-suppressive properties like down-regulation of oncogene activity, apoptosis, invasion, and metastasis [25]. Thus any change in *FHIT* protein expression might result in a number of changes at molecular level and hence it’s altered functions ultimately lead to different kinds of diseases including tumorogenesis [26]. Reports are also available regarding the *FHIT* gene hypermethylation as an important contributor in causation of different malignancies [27]. Because epigenetic modifications can have significant effect on transcriptional activity in candidate tumor suppressor genes, it can promote tumorogenesis including leukemia’s [28, 29].

### FHIT gene mRNA expression and its association with FHIT promoter hyper-methylation

Recent advances in the molecular studies have led to deep understanding of ALL and a number of studies have provided evidence for the constitutive activation of *FHIT* gene and its association with ALL [16, 17]. Altered expression of *FHIT* is one of the potential mechanisms that promote tumor progression and its decreased expression is associated with signaling pathways in various cancers [30].

In the present study, the prognostic significance of *FHIT* expression in ALL patients was evaluated for *FHIT* hypermethylation. The mRNA expression of *FHIT* analyzed in ALL cases associated with a reduced pattern of expression. Significantly decreased expression was observed in majority of ALL cases (75%) than healthy controls (p = 0.01). The findings of the current study with *FHIT* mRNA down regulation clearly demonstrated the plausible involvement of *FHIT* gene expression to play an important role in the pathogenesis of ALL development. In a study by Hallas et al. [22] that confirms and agree with our report, loss of *Fhit* protein expression was detected in a majority of primary ALL cases and leukemia cell lines. Our results were in accordance with Chen et al. [31], where the expression of *FHIT* mRNA or protein was reported to be altered in 70% of cases. Further, Malak et al. [32] also agreed with our results wherein significantly less expression of *FHIT* mRNA was demonstrated for childhood ALL. In yet another report, Iwai et al*.* [21] demonstrated the RNA expression of the *FHIT* gene in cases of leukemia (ALL n = 11) and acute myeloid leukemia (n = 40). These results were consistent with our study and showed *FHIT* gene expression was totally lost or significantly reduced in 46% of the ALL cases and even 55% AML cases [33]. This also signifies the diverse role of *FHIT* in different types of leukemia. Reduced or loss of *FHIT* expression has been confirmed in solid tumors like head and neck, GIT, renal cell, breast, cervical cancers and others [34–36]. It has been substantiated by Siprashvili et al. [37] that transfection of *FHIT* into tumorigenic cell lines reduces tumor process in nude mice; pointing to the fact that *FHIT* acts as a tumor suppresser gene. Studies have observed that dysregulation or loss of *FHIT* mRNA expression occurs frequently in various cancers [38–40] but loss of the *Fhit* protein has been seen in various hematological malignancies as well [40]. Substantial evidence from a few studies suggested that the alteration of normal *FHIT* function could be an important event in the pathogenesis of many human hematological malignancies including ALL and the altered *FHIT* gene expression is specifically identified factor and highly persistent event in leukemia [21, 41]. The frequent alteration in particular reduction in *FHIT* expression as found in this study in ALL and other quoted studies gives an evidence that inactivating changes at the *FHIT* locus could supplement for the development to leukemia’s [22]. When the results of the studies are taken together with our report where clinical sample of ALL were directly examined for *FHIT* expression, the results demonstrate that dysregulation of *FHIT* expression has a plausible link in the pathogenesis of ALL. Evidences show that numerous tumor suppressor genes are inactivated by promoter region methylation in different malignancies like p1*5INK4B* gene was frequently inactivated by promoter methylation in myelodysplastic syndrome and AML [42]. Similarly *FHIT* gene methylation has been depicted in few solid tumors, like GIT tumors, lung and breast cancers, where it was linked to dysregulated expression [43, 44]. Further, loss or reduced expression of *FHIT* protein due to its altered methylation was shown to be connected to progression of disease in certain malignancies. [45, 46] In the current study, the results showed *FHIT* mRNA expression was significantly decreased in those ALL cases that exhibited with hypermethylated *FHIT* sequences (*p* = 0.0008). In concordance to our results, study by Bahari et al. [47] showed that the *FHIT* gene hypermethylation was significantly abundant in ALL cases with *FHIT* expression seen significantly lower in ALL patients.

The genetic and epigenetic modification in critical regulatory genes may affect gene expression or the structure and function of specific gene products which can lead to leukemia development. In this investigation, hypermethylation of the *FHIT* gene was found to be considerably greater in ALL patients than in healthy controls. Our findings show that epigenetic changes and abnormal gene expression of *FHIT* in ALL patients may play a role in the disease’s progression. Consistent with our findings, Malak et al. [32] found a substantial decrease in the expression of *FHIT* in ALL compared to healthy controls. Furthermore, another study demonstrated that when compared to controls, ALL samples had significantly lower expression of *FHIT* with an increase in methylation frequency [31]. The association becomes crucial according to several studies as DNA methylation is the most often identified change in individuals with ALL. Further, *FHIT* gene deregulation in particular the decreased expression due to promoter gene methylation has been related to solid tumors for disease progression [33, 48]. In accordance to our report, a study by Hallas et al. [22] established the role of *FHIT* gene alteration in ALL where loss of *FHIT* mRNA and its product in majority of cases in contrast to their presence in almost all healthy control samples specified its plausible contribution to the pathogenesis ALL. *FHIT* gene as reported is emerging as a putative therapeutic target not only for ALL but other cancer types as well. A meta-analysis conducted by Wu et al*.* [16] where it was shown that the inactivation of *FHIT* gene by hypermethylation is associated with an elevated risk of non-small cell lung cancer. Therefore, the aberrant methylation patterns along with decrease in the expression of *FHIT* observed as seen in our study and other ones cannot be interpreted as simple secondary event, but rather must play a critical role in determining the malignant phenotype.

## Conclusion

The current study advocates that *FHIT* gene hypermethylation and decreased mRNA may be linked in the pathogenesis of ALL and provide an evidence for the role of *FHIT* in development of ALL. However, a better interpretation of *FHIT* methylation and its expression may be a way forward to gain knowledge into epigenetic mechanisms of ALL to devise a strategy for treatment and unravel its different facets for chemotherapeutic outcome.

### Supplementary Information

**Additional file 1. **Supplementary Figure.

## Data Availability

The data supporting the results of this study are available within the paper and it’s Supplementary Information.
