# Supplementary material for: Significance and implications of FHIT gene expression and promoter hypermethylation in acute lymphoblastic leukemia (ALL)
Source: Discov Oncol. 2024 Apr 8;15:108. doi: 10.1007/s12672-024-00971-9 (PMC11001825; doi:10.1007/s12672-024-00971-9)
Supplement: Supplementary file 1 — Additional file 1. Supplementary Figure. [file 12672_2024_971_MOESM1_ESM.zip › New folder/Supplementary_figs.docx]

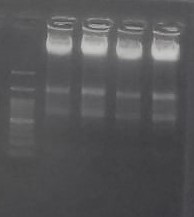


**28s rRNA**

**18s rRNA**

***Supplementary Figure1: Representative gel picture of RNA isolated from blood, of ALL patients.***

**
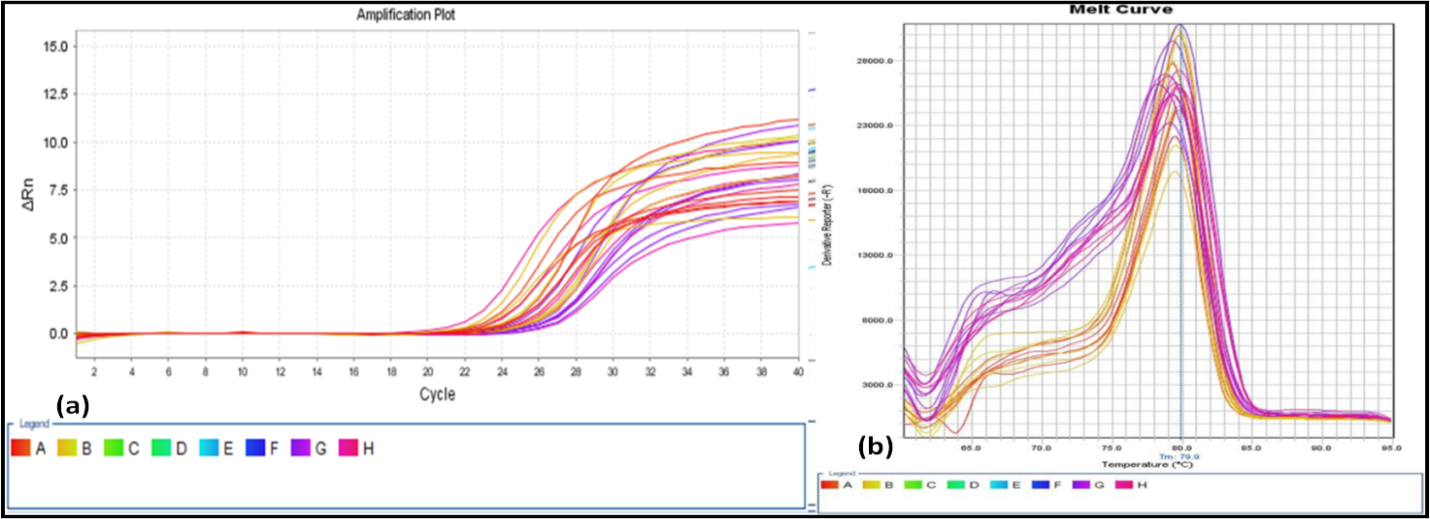
**

Supplementary Figure 2: Representative amplification plots (a) and melt curve (b) of FHIT and GAPDH gene.


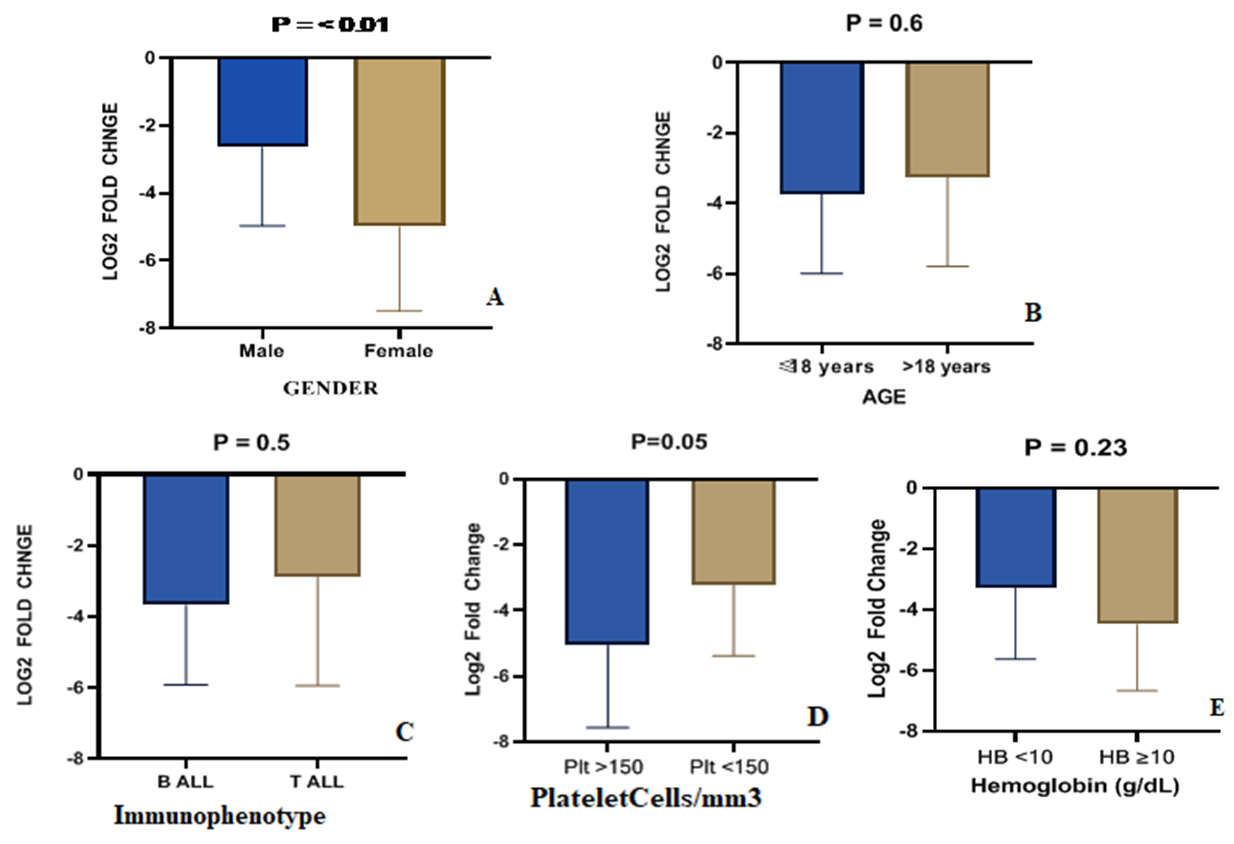
Supplementary Figure 3: *Comparison of log2 fold change expression of FHIT gene with clinical parameters*. *(A) represents FHIT gene expression vs. gender; (B) FHIT gene expression vs. Age; (C) FHIT gene expression vs. Immunophenotype (D) FHIT gene expression vs. platelet count (E) FHIT gene expression vs. Hemoglobin (g/dl)*
