# Supplementary material for: Significance and implications of FHIT gene expression and promoter hypermethylation in acute lymphoblastic leukemia (ALL)
Source: Discov Oncol. 2024 Apr 8;15:108. doi: 10.1007/s12672-024-00971-9 (PMC11001825; doi:10.1007/s12672-024-00971-9)
Supplement: Supplementary file 1 — Additional file 1. Supplementary Figure. [file 12672_2024_971_MOESM1_ESM.zip › New folder/Supplementary_Tables (1).docx]

Supplementary Table S1: Primer sequences and annealing temperatures of GAPDH and FHIT gene for qRT-PCR

| **Gene** | **Primer Sequence** | **Annealing temperature** | **Base Pair (bp)** |
| --- | --- | --- | --- |
| **GAPDH –RT** | F: 5ʹ- GAAGGTGAAGGTCGGAGTC -3ʹ  R: 5ʹ- GAAGATGGTGATGGGATTTC-3ʹ | 63*˚C* | 226 |
| **qRT-PCR**  **FHIT-RT** | F: 5ʹ- ACCTGCGTCCTGATGAAGTG-3ʹ  R: 5ʹ- CGTGAACGTGCTTCACAGTC-3ʹ | 63*˚C* | 144 |
| FHIT Methylated primers | F: 5ʹ- TTTTCGTTTTTGTTTTTAGATAAGC-3´  F: 5ʹ- AAAAATATACCCACTAAATAACCGC-3ʹ | *58˚C* | 157 |
| FHIT Unmethylated primers | F: 5ʹ- TGGTTTTTGTTTTTGTTTTTAGATAAGT-3´  F: 5ʹ- AAAATATACCCACTAAATAACCACC-3´ | *58˚C* | 159 |
| ***F= forward primer; R= reverse primer;*** | | | |
